# TGFβ signaling links early-life endocrine-disrupting chemicals exposure to suppression of nucleotide excision repair in rat myometrial stem cells

**DOI:** 10.21203/rs.3.rs-3001855/v1

**Published:** 2023-06-07

**Authors:** Maria Victoria Bariani, Yan-Hong Cui, Mohamed Ali, Tao Bai, Sandra L. Grimm, Cristian Coarfa, Cheryl L. Walker, Yu-Ying He, Qiwei Yang, Ayman Al-Hendy

**Affiliations:** University of Chicago Department of Obstetrics and Gynecology; University of Chicago Department of Medicine; University of Chicago Department of Obstetrics and Gynecology; University of Chicago Department of Obstetrics and Gynecology; Baylor College of Medicine; Baylor College of Medicine; Baylor College of Medicine; University of Chicago Department of Medicine; University of Chicago Department of Obstetrics and Gynecology; University of Chicago Department of Obstetrics and Gynecology

**Keywords:** Uterine fibroid risk, myometrial stem cells, endocrine-disrupting chemicals, transforming growth factor beta, nucleotide excision repair, developmental reprogramming

## Abstract

Environmental exposure to endocrine-disrupting chemicals (EDCs) is linked to the development of uterine fibroids (UFs) in women. UFs, non-cancerous tumors, are thought to originate from abnormal myometrial stem cells (MMSCs). Defective DNA repair capacity may contribute to the emergence of mutations that promote tumor growth. The multifunctional cytokine TGFβ1 is associated with UF progression and DNA damage repair pathways. To investigate the impact of EDC exposure on TGFβ1 and nucleotide excision repair (NER) pathways, we isolated MMSCs from 5-months old Eker rats exposed neonatally to Diethylstilbestrol (DES), an EDC, or to vehicle (VEH). EDC-MMSCs exhibited overactivated TGFβ1 signaling and reduced mRNA and protein levels of NER pathway components compared to VEH-MMSCs. EDC-MMSCs also demonstrated impaired NER capacity. Exposing VEH-MMSCs to TGFβ1 decreased NER capacity while inhibiting TGFβ signaling in EDC-MMSCs restored it. RNA-seq analysis and further validation revealed decreased expression of Uvrag, a tumor suppressor gene involved in DNA damage recognition, in VEH-MMSCs treated with TGFβ1, but increased expression in EDC-MMSCs after TGFβ signaling inhibition. Overall, we demonstrated that the overactivation of the TGFβ pathway links early-life exposure to EDCs with impaired NER capacity, which would lead to increased genetic instability, arise of mutations, and fibroid tumorigenesis. We demonstrated that the overactivation of the TGFβ pathway links early-life exposure to EDCs with impaired NER capacity, which would lead to increased fibroid incidence.

## Introduction

The incidence of uterine fibroids (UFs) is extremely common among women of reproductive age. Although non-cancerous, these tumors are associated with significant morbidity, including prolonged or heavy menstrual bleeding, pelvic pain, and in some cases, they can be related to pregnancy loss and infertility [1]. Despite the last few years have shown emergence of different treatment alternatives, UFs continue to be the most common cause of hysterectomy, which in turn increases these women’s risk for several medical complications [2].

Risk factors for the development of these tumors include race/ethnicity, age, parity, and Vitamin D deficiency [1]. Notably, endocrine-disrupting chemicals (EDCs), both natural and anthropogenic, are capable of disrupting the endocrine system, and may contribute to some of the most prevalent female reproductive disorders [3, 4]. In this sense, environmental EDCs exposure is considered an important risk factor for UF pathogenesis [5, 6]. Moreover, epidemiological studies have confirmed the correlation between early life exposure to EDCs and increased risk for early UF diagnosis [7–10]. However, to understand how the exposure to EDCs during critical uterine developmental period could increase the incidence of UFs is yet unclear and necessary to appeal to animal experimentation, and the best-characterized animal model for this is the Eker rat. These animals carry a defect in the *Tsc2* tumor suppressor gene and female Eker rats develop UFs spontaneously with a high frequency during their adulthood [11]. Cook et al. [12] have shown that early-life exposure to diethylstilbestrol (DES) during the development of the uterus increased *Tcs2* penetrance, tumor multiplicity and size, demonstrating that developmental exposure to EDCs can permanently reprogram tissue responses. Even though DES is not currently in use, it is an effective tool to study the effects induced by this EDC since many environmental xenoestrogens such us dibutyl phthalate (DBP) have a similar impact on reproductive health [13].

Several studies support the argument that UFs originate from transformed myometrial stem cells (MMSCs) [14–16]. DNA damage response and repair processes maintain the integrity of genomic DNA, and failures in these mechanisms may be the cause behind the transformation of a normal MMSC to a tumor initiating cell [17, 18]. In this regard, EDCs can cause DNA damage upon exposure [19, 20]. Previous observations from our group have indicated that the expression of DNA repair-related genes/proteins is reprogrammed by early-life EDCs exposure in MMSCs isolated from adult Eker rats [21, 22]. Nucleotide excision repair (NER) is a highly conserved DNA repair pathway, capable of removing structurally bulky DNA helix distortion lesions from the genome, generated by chemicals or UV radiation [23, 24]. To date, there is limited available information regarding the potential inference of the NER pathway on the physiopathology of UF. A single report done in southern Chinese women has linked a higher susceptibility to UF with a single nucleotide polymorphism in *XPG* gene (rs873601 G > A), a crucial protein involved in NER repair processes [25].

Transforming growth factor β (TGFβ) is a secreted cytokine that exists in mammals in three isoforms (TGFβ1, TGFβ2, and TGFβ3). TGFβ controls a plethora of processes, such as apoptosis, angiogenesis, and tumor biology [26], and is considered one of the key factors in the pathophysiology of UFs [27]. Several studies have suggested a connection between TGFβ signaling and DNA damage response [28, 29]. Previous analyses reported a link between TGFβ1 pathway and steroid hormone signaling [30, 31] or EDCs treatment [32, 33].

This work aimed to elucidate the role of both TGFβ1 and the NER, as well as their link to the tumorigenesis process on MMSCs, the cell origin of UFs, in the best-characterized animal model of UFs for gene-environment interaction.

## Material and Methods

### Animal model and Myometrial Stem Cell isolation and culture

Female Eker rats from an on-site colony (Long Evans; *TSC-2*^Ek/+^) received subcutaneous injections of 10 μg of the endocrine-disrupting chemical (EDC) Diethylstilbestrol (DES, D4628, Sigma, St. Louis, MO, USA) per rat per day or 50 μl of sesame seed oil (vehicle, VEH, S3547, Sigma, St. Louis, MO, USA) on 10, 11, and 12 postnatal days, a sensitive period for uterine developmental programming, as previously described [12]. Animals were euthanized at 5 months of age and subjected to myometrial Stro1+/CD44 + stem cell isolation, according to a previously described protocol [80]. Briefly, uterine tissues from Eker rat exposed to VEH (N = 5, pooled) or EDC (N = 5, pooled) were collected, washed to remove residual blood, and the endometrial and serosal tissues were removed by scraping with a sterile scalpel. Myometrial tissues were digested into single-cell suspensions, which were subjected to selection for Stro1/CD44 double-positivity by magnetic beads (mouse anti-Stro1, MAB1038, R&D Systems and mouse anti-CD44, #555478, BD Biosciences, respectively) to isolate Stro1+/CD44 + MMSCs. Then, isolated VEH- and EDC-MMSCs were plated in coated flasks (Attachment factor #S006100, Thermo Fisher Scientific, Waltham, MA) and cultured separately in Smooth Muscle Growth Medium-2 BulletKit (complete SmGm media) (CC-3182, Lonza, Walkersville, MD) under hypoxic conditions (37°C, 5% CO_2_, 2% O_2_). Confluent VEH- and EDC-MMSCs were washed with PBS, trypsinized (TrypLE Express Enzyme, 12604021, Thermo Fisher Scientific, Waltham, MA), and centrifuged at 500 × g for 5 min. Supernatants were aspirated, and pellets were stored at −80°C until further use. Protocols involving the use of these animals were approved by the Institutional Animal Care & Use Committee (IACUC), Baylor College of Medicine (protocol # AN-7189).

### RNA isolation, cDNA synthesis, and quantitative real-time PCR

Total cellular RNA was isolated from frozen MMSCs pellets using TRIzol Reagent (#15596026, Invitrogen, Waltham, MA, USA) following manufacturer instructions. RNA reverse transcription to complementary DNA (cDNA) was performed using Ecodry premix double-primed (#639549, Takara Bio, San Jose, CA, USA). Quantitative real-time PCR (qPCR) was carried out using SsoAdvanced Universal SYBR Green Supermix (Bio-Rad, Hercules, CA, USA) in a 20-μL final reaction volume. Primer sequences are listed in Table 1. Primers were purchased from Integrated DNA Technologies (IDT, Coralville, IA, USA) excluding *Ltbp1, Tgfb1, Smad3*, and *Uvrag* primers (Product IDs: RQP050189, RQP050181, RQP090103, RQP083172, respectively) that were purchased from Genecopoeia (Rockville, MD, USA). Real-time PCR analyses were performed using the Bio-Rad CFX96 detection system (Bio-Rad, Hercules, CA, USA). A melting-curve analysis affirmed the synthesis of a DNA product of the predicted size. The expression data were normalized using *18S* ribosomal RNA values, and these relative normalized values were used to generate data graphs. A reaction without a cDNA template was used as a negative control.

### Protein expression analysis by western blot

VEH- and EDC-MMSCs pellets were lysed in RIPA buffer (#89900, Thermo Fisher Scientific, Waltham, MA) containing 1% of protease and phosphatase Inhibitor Cocktail (#78440, Thermo Fisher Scientific, Waltham, MA), vortexed, sonicated, and centrifuged for 10 min at 12,000 RPM at 4◦C. Three experimental replicates per group were run. Samples equivalent to 25 μg of protein were separated using 4–20% Mini-PROTEAN TGX Precast Protein Gels (#4561096, Bio-Rad, Hercules, CA) and transferred to Trans-Blot Turbo Midi 0.2 μm PVDF membranes (#1704157, Bio-Rad, Hercules, CA) according to standard procedures. Membranes were blocked for 1 h at RT in either 5% w/v nonfat dry milk or 5% BSA in 0.1% Tween-supplemented PBS (0.1% PBS-T) per antibody specification. Membranes were then incubated with primary antibodies overnight at 4◦C in either 1% w/v nonfat dry milk or 1% BSA in 0.1% PBS-T per antibody specification. Following is the information regarding the primary antibodies used, their source, and working dilutions: rabbit anti-LTBP1 (ab78294, Abcam; 1:1000), rabbit anti-THBS1 (MA5–13398, Invitrogen; 1:1000), rabbit anti-TGFβ 1 (MA5–15065, Invitrogen; 1:1000), rabbit anti-p-SMAD2 (Ser465/467) (#3108, Cell Signaling; 1:1000), rabbit anti-SMAD2 (#5339, Cell Signaling; 1:1000), rabbit anti-XPA (PA5–86265, Invitrogen; 1:1000), mouse anti-XPB (#8746, Cell Signaling; 1:1000), mouse anti-XPC (sc-74410, Santa Cruz; 1:1000), rabbit anti-XPD (#11963, Cell Signaling; 1:1000), rabbit anti-XPG (PA5–76039, Invitrogen; 1:1000), rabbit anti-XPF (#13465, Cell Signaling; 1:1000), rabbit anti-DDB1 (#5428, Cell Signaling; 1:1000), mouse anti-DDB2 (sc-81246, Santa Cruz; 1:1000). Mouse anti-β-Actin (A5441, Sigma, 1:10000) protein levels were assessed by re-probing the blots. Membranes were washed in 0.1% PBS-T and then incubated with anti-rabbit (#7074, Cell Signaling; 1:5000) or anti-mouse (#7076, Cell Signaling; 1:5000) horseradish peroxidase-labeled antibodies. The antigen–antibody complex was detected with Trident femto Western HRP Substrate kit (GTX14698, GeneTex, Irvine, CA, USA) and images of immunoreactive bands were acquired using ChemiDoc XRS + molecular imager (Bio-Rad, Hercules, CA, USA). Bands were analyzed using Image J software [81]. The relative protein level was normalized to β-actin and results were expressed as relative optical density.

### Measurement of TGFβ1 levels in MMSCs culture supernatants

VEH- and EDC-MMSCs were cultured until confluence in previously stated conditions. Then, cells were washed thoroughly using PBS, and media were replenished with complete SmGm media without fetal bovine serum. MMSCs culture supernatant (CS) samples were collected after 6 h and frozen in aliquots at − 80°C. TGFβ1 levels were detected in CS using a solid phase ELISA kit (DB100C, R&D Systems, Minneapolis, MN, USA) according to the manufacturer’s protocol.

### Immunofluorescence

*VEH- and EDC-MMSCs* were seeded onto coated-glass coverslips and culture under the conditions stated above. Confluent cells were then fixed with 4% paraformaldehyde for 15 minutes, followed by permeabilization with 0.1% Triton X-100 in PBS for 15 minutes at room temperature. Non-specific binding was blocked with 2% BSA in PBS for 1 hour at room temperature. Primary antibody targeting TGFβ1 (MA5–15065, Invitrogen, 1:100 in 0.1% BSA-PBS) and TGFβ Receptor I (PA-95863, Invitrogen, 1:100 in 0.1% BSA-PBS) were applied and incubated overnight at 4°C. The cells were then washed with PBS three times for 5 min and incubated with Alexa Fluor^™^ 568-conjugated α-Rabbit secondary antibody (A11011, Invitrogen, 1:2000 in 0.1% BSA-PBS) for 1 hour at room temperature. Finally, the coverslips were mounted onto glass slides using mounting medium containing DAPI for nuclear counterstaining (H-1200, VECTASHIELD). Digital image files were created with an Olympus VS200 Research Slide Scanner (Olympus / Evident, Center Valley, PA) with a Hamamatsu ORca-Fusion camera (Hamamatsu Photonics, Skokie, IL). Individual images were created with the OlyVIA Viewer software (Olympus / Evident, Center Valley, PA). Negative controls without primary antibody were included to validate the staining specificity.

### Immunohistochemistry

Myometrial tissue samples from 5 months old Eker rat exposed neonatally to VEH or EDC were fixed in 10% buffered formalin for 15–20 h and embedded with paraffin. Paraffin blocks were sliced into 5-μM thick sections, deparaffinized with xylene, and rehydrated by being passed through decreasing concentrations of ethanol in water. Then, antigen retrieval and quenching of endogenous peroxidases were performed. The primary antibodies used to detect XPA and XPC were rabbit anti-XPA (PA5–86265, Invitrogen; 1:250) and mouse anti-XPC (sc-74410, Santa Cruz; 1:200), respectively. Samples were scanned and visualized using Aperio Image Scope Software (v12.4.0.7018) (Leica Biosystems Imaging Inc., Deer Park, IL, USA).

### MMSCs TGFβ1 and TGFβ Receptor I inhibitor treatments

Once VEH- or EDC-MMSCs reached 80% confluence, they were treated with human recombinant TGFβ1 (10 ng/ml, 7754-BH, R&D Systems,) for 48 h or with TGFβ Receptor I inhibitor (2μM, LY-364947, L6293, Sigma, St. Louis, MO, USA) for 24 h, respectively. The vehicle used to dissolve TGFβ1 was 4 mM HCl (SA49, Thermo Fisher Scientific, Waltham, MA, USA) containing 0.1% bovine serum albumin (BSA, A3294, Sigma, St. Louis, MO, USA). Mature human TGFβ1 shares 99% amino acid identity with rat TGFβ1, and it demonstrated cross-species activity [82]. Dimethyl sulfoxide (DMSO, 472301, Sigma, St. Louis, MO, USA) was used as a VEH to dissolve the TGFβ Receptor I inhibitor (final concentration < 0.1%). VEH- and EDC-treated MMSCs were washed with PBS, trypsinized, and centrifuged at 500 × g for 5 min. The supernatant was discarded, and the pellets were snap-frozen and stored at −80°C for RNA isolation.

### Determination of UVB-induced DNA damage in genomic DNA by slot blot assay

VEH- and EDC-MMSCs pellets were collected as described above at different time points (0, 6, and 12 h) post-UVB light exposure (10 mJ/cm^2^), and DNA was isolated using a QIAamp DNA Mini Kit (#51304, Qiagen, Valencia, CA). The DNA concentration was calculated from the absorbance at 260 nm using NanoDrop 1000 (NanoDrop products, Wilmington, DE). The cyclobutane pyrimidine dimers (CPD) in DNA were quantified by slot blot (Bio-Rad) with CPD monoclonal (TDM-2) antibody (CAC-NM-DND-001, COSMO BIO Co., Koto-Ku, Tokyo, Japan) as described previously [83]. The chemiluminescence was detected with a Carestream Imaging Station (Carestream, Rochester, NY, USA). For examining repair kinetics, the percentage (%) of CPD repair was calculated by comparing the optical density at the indicated time to that of the corresponding absorbance at time zero when there was no opportunity for repair, and 100% of CPDs were present post-UVB. The 10 mJ/cm^2^ UVB dose was chosen as there was little acute, UV-induced cell death (observed under light bright microscopy) induced under these conditions, while there were sufficient levels of DNA damage to reproducibly measure its repair (doses between 10 ~ 30 mJ/cm^2^ were screened initially).

### Whole-genome RNA sequencing (RNA-seq)

RNA quality and quantity were assessed using the Agilent bio-analyzer. Strand-specific RNA-SEQ libraries were prepared using a TruSEQ mRNA-SEQ library protocol (Illumina provided). Library quality and quantity were assessed using the Agilent bio-analyzer, and libraries were sequenced using an Illumina NovaSEQ6000 (Illumina provided reagents and protocols). A variety of R packages were used for this analysis. All packages used are available from the Comprehensive R Archive Network (CRAN), Bioconductor.org, or Github. The reads were mapped to the *R. norwegicus* reference genome Rnor 6.0 using STAR 2.7.9a. Aligned were quantified using Salmon 1.4.0, and gene annotations from Ensembl were used to summarize data from transcript level to gene level. We filtered non-protein coding genes as well as genes with less than 1 count per million in at least 3 or more samples and applied TMM normalization. To identify differentially expressed genes (DEGs), precision weights were applied to gene counts based on within-group sample-level variance and gene-level mean-variance trends using VOOM from Limma 3.52.4. The count data was fitted to a gene-wise linear model with group status as a coefficient, and an empirical Bayes method was used to estimate the posterior odds of differential expression after adjusting for gene-level posterior residual standard deviations. Significant differential genes were decided with a minimum absolute fold change of 1.5 and a false-discovery rate of 0.05. GSEA was tested using the Hallmark MSigDB collection.

### Statistical analysis

Comparisons between groups were made by two-tailed unpaired Student’s t-test using GraphPad Prism 9 (GraphPad Software, San Diego, CA). The assumption of normality was assessed by Shapiro–Wilks test. All data are presented as mean ± standard error of mean (S.E.M.). A difference between groups with *p < 0.05, **p < 0.005, ***p < 0.0005, or ****p < 0.0001 was considered statistically significant.

## Results

### The TGFβ1 signaling pathway is overactivated in MMSCs isolated from rats exposed to EDCs

**Figure 1:**
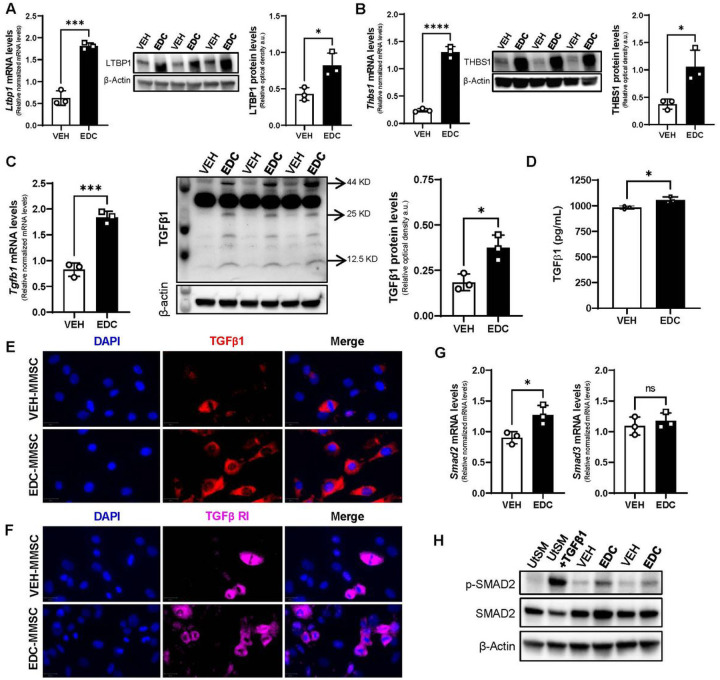
The TGFβ1 pathway is overactivated in MMSCs isolated from rats neonatally exposed to EDC. Real-time PCR analysis of mRNAs, protein levels and representative gels of **A**) LTBP1, **B**) THBS1, and **C**)TGFβ1 (arrows indicate band for: Pro-TGFβ1 at 44 kDa, Dimer of mature TGFβ1 at 25kDa, and Monomer of mature TGFβ1 at 12.5kDa) in VEH- and EDC-MMSCs isolated from 5-months old rats. **E**) TGFβ1 levels in culture supernatants collected from VEH- and EDC-MMSCs cultures. Immunofluorescence images of **D**) TGFβ1 and **F**) TGFβ1 RI in VEH- and EDC-MMSCs. Scale bar= 20 μm. **F**) mRNAs levels of *Smad2, and Smad3* in VEH- and EDC-MMSCs isolated from 5-months old rats. **G**) Representative gel of p-Smad2 and Smad2 in VEH- and EDC-MMSCs. mRNA data were normalized by the amount of 18S and protein levels by the amount of β-Actin. Data are shown as mean ± S.E.M. from triplicate data. ns= not significant. *= p< 0.05, ***= p< 0.001, ****= p<0.0001, Student’s t-test. EDC: endocrine disrupting chemical, MMCSs: myometrial stem cells, THBS1: Thrombospondin 1; LTBP1: Latent TGFβ binding protein 1. TGFβ1: Transforming growth factor beta 1. TGFβ RI: Transforming growth factor beta receptor 1. UtSM: human uterine smooth muscle cell line. TGFβ1 treatment: 10 ng/ml for 1h (positive control)

To identify transcriptional changes in the context of early-life EDCs exposure, we performed RNA-seq on MMSCs isolated from 5-months old Eker rats exposed neonatally to VEH or to diethylstilbestrol (DES), an EDC that mimic estrogen action [34]. We found 2922 DEGs (1474 up, and 1448 down) in EDC- over VEH-MMSCs (Figure S1 A). Among the DEGs, we found 14 genes belonging to TGFβ signaling (Figure S1 B). In addition, pathway analysis using Hallmark compendium showed significant enrichment of the HALLMARK_TGF_BETA_SIGNALING pathway (Figure S1 C). There is evidence that perinatal exposure to the estrogenic EDC methoxychlor reprogramed *Tgfb1* gene expression in the hypothalamus of Fischer rats [35]. Moreover, Cometti et al. have reported that certain environmental estrogen induced TGFβ1 levels in bovine oviduct cell culture [36], linking steroid hormone signaling with TGFβ1. In this study, to determine whether early life exposure to EDCs affected TGFβ1 pathway on rat MMSCs, we first analyzed several members of this superfamily. We found that the mRNA and protein levels of latent TGFβ binding protein 1 (LTBP1), which controls TGFβ1 bioavailability by maintaining it in a latent state in the extracellular matrix [37], were increased in EDC-MMSCs compared to VEH-MMSCs (Fig. 1A). Further, we observed the same outcome for thrombospondin-1 (TSP1), a major regulator of latent TGFβ1 activation [38] (Fig. 1B). We further confirmed that TGFβ1 mRNA and protein levels are also elevated in MMSCs isolated from rats exposed to EDCs in early life (Fig. 1C and E). Moreover, as illustrated in Fig. 1D, the levels of TGFβ1 were significantly higher in the culture supernatants from EDC-MMSCs compared to the ones from VEH-MMSCs. Additionally, we confirmed the presence of TGFβ Receptor I on VEH- and EDC-MMSCs using immunofluorescence staining (Fig. 1F). SMADs are the main transducers of the TGFβ superfamily signal from the cell surface to the nucleus [39]. We found that the mRNA and protein levels of SMAD2 and p-SMAD2, which is considered a marker of TGFβ signaling activation, are increased respectively in EDC-MMSCs compared to the control (Fig. 1G and H) while *Smad3* (Fig. 1G) mRNA levels did not change. Overall, these results show that the TGFβ1 pathway is overactivated in MMSCs isolated from rats developmentally exposed to EDCs.

### Early life exposure to EDCs provokes changes in MMSC NER pathway members

**Figure 2:**
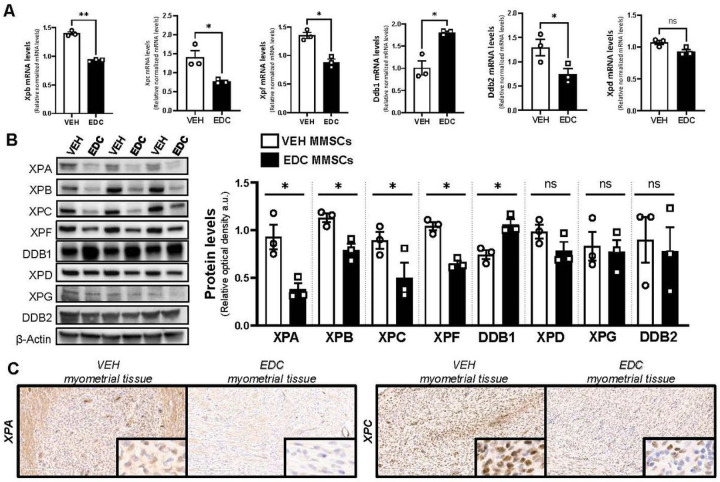
Characterization of nucleotide excision repair (NER) pathway in VEH- and EDC-MMSCs. **A**) mRNA levels of *Xpb, Xpc, Xpf, Ddb1, Ddb2* and *Xpd* in VEH- and EDC-MMSCs isolated from 5-months old rats. 18s were used to normalize the expression data. **B**)Representative gel and protein levels of XPA, XPB, XPC, XPF, DDB1, XPD, XPG, and DDB2 in VEH- and EDC- MMSCs isolated from 5-months old rats. Data were normalized by the amount of β-Actin protein levels. **C**) IHC images (20X magnification, insets are at 40X magnification) of XPA and XPC in myometrial tissues from 5-months old Eker rats treated neonatally with VEH or EDC. Scale bar = 200 μm. Data are shown as mean ± S.E.M. from triplicate data. * = p< 0.05, **= p< 0.01, Student’s t-test. EDC: endocrine disrupting chemical, MMSCs: myometrial stem cells, XP: Xeroderma pigmentosum, DDB1/2: DNA damage-binding protein ½

The analysis of EDC- and VEH-MMSC RNA-seq data demonstrated significant enrichment of the HALLMARK_UV_RESPONSE pathways along with enrichment of other pathways related to DNA damage repair such us HALLMARK_G2M_CHECPOINT (Figure S1 C). Moreover, 6 genes involved in nucleotide excision repair (NER) pathway showed changes in their mRNA expression levels between EDC-MMSCs and VEH-MMSC (Figure S1 D). NER is the main pathway involved in repairing bulky DNA adducts formed by environmental carcinogenic sources such as UV light exposure or chemical agents [40]. DES, the EDC used in this work as a research tool, is metabolized to reactive intermediates that covalently bind to DNA and nuclear proteins forming adducts [41]. To examine whether the factors that execute NER are regulated in MMSCs by environmental EDC exposure, we evaluate the mRNA and protein levels (Fig. 2A and B, respectively) of several core enzymes involved in different steps during the NER process. The damage sensor XPC exhibited lower mRNA and protein levels in EDC-MMSCs contrasted with VEH-MMSCs. Interestingly, when we evaluated DDB1 and DDB2 (DNA damage-binding protein 1 and 2), which also play central roles in the damage recognition process, we found that DDB1 mRNA and protein levels increased in EDC-MMSCs compared to the control. In contrast, DDB2 (also known as XPE) showed decreased mRNA levels on MMSCs isolated from EDC-exposed rats compared to controls but we did not see any differences in its protein levels. A previous study from our group have demonstrated that the mRNA levels of *Xpa*, which functions as a scaffold to assemble other NER core factors around the DNA damage site [42], were significantly downregulated in EDC-MMSCs compared with VEH-MMSCs [22]. In this work, we showed that the protein levels of XPA presented the same outcome (Fig. 2B). The DNA helicase XPB showed decreased mRNA and protein levels in EDC- versus VEH-MMSCs. However, we did not find any difference in the DNA helicase XPD mRNA or protein levels. XPF provides the endonuclease activity in a heterodimer complex that is essential for repairing DNA damage [43]. We observed that XPF mRNA and protein levels decreased in EDC-MMSCs in comparison to VEH-MMSCs. In conclusion, these results demonstrate that early life exposure to EDCs provokes changes in several NER pathway members in rat MMSCs, mostly, decreasing their levels.

MMSCs present self-renewal and differentiation capacities which are critical for myometrial tissue homeostasis. To evaluate whether the observed changes in NER pathway members take place also at the tissue level, we performed XPA and XPC IHC in myometrial tissues collected from adult rats (pre-fibroid) exposed neonatally to VEH or EDC (Fig. 2C). We found that the levels of both markers were lower in the myometrium of EDC-exposed rats compared to controls, suggesting that differentiated myometrial cells maintain characteristics of the parent MMSCs.

### EDC-MMSCs present decreased ability to repair DNA by NER

**Figure 3:**
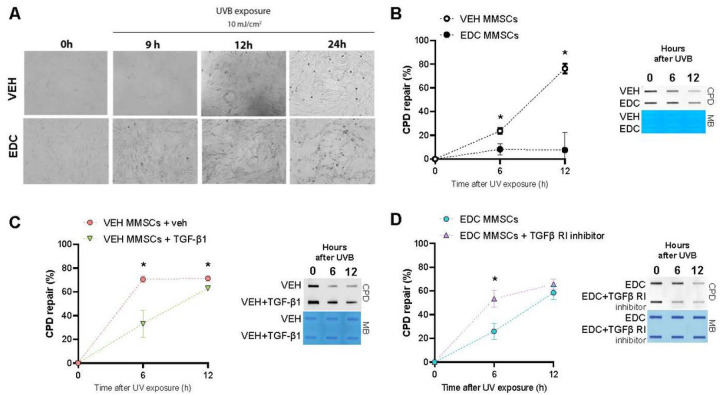
Effect of early life EDC exposure and TGFβ1 on CPD repair. **A**)Bright-field images of VEH- and EDC-MMSCs before (0h) and after UVB exposure (9, 12, and 24 h; 10 mJ/cm2). Magnification 20X. **B**) Quantification of percentage (%) of CPD repair and a representative image of DNA slot blot in VEH- and EDC-MMSCs isolated from 5 months old rats at 0, 6 and 12 h post-UVB (10 mJ/cm2). **C**) Quantification of percentage (%) of CPD repair and a representative image of DNA slot blot in VEH-MMSC treated with vehicle (4 mM HCl + 0.1% BSA) or TGFβ1 (10 ng/ml) for 48 h and then collected at 0, 6 and 12 h post-UVB (10 mJ/cm2). **D**)Quantification of percentage (%) of CPD repair and a representative image of DNA slot blot in EDC-MMSC treated with vehicle (<0.1% DMSO) or TGFβ1 Receptor inhibitor (2μM) for 24 h and then collected at 0, 6 and 12 h post-UVB (10 mJ/cm2). Methylene blue (MB) was used as the loading control. Data are shown as mean ± S.E.M. from triplicate data. *= p< 0.05, Student’s t-test

To determine whether early life exposure to EDCs affects NER repair capability in MMSCs, we assessed the repair kinetic of cyclobutane pyrimidine dimers (CPD) after UVB exposure in VEH- and EDC-MMSCs. Cells were irradiated with 10 mJ/cm^2^ UVB light and the cellular morphology were monitored by phase contrast light microscopy before and 9,12 and 24 h after the exposure. Figure 3A illustrates that EDC-MMSCs revealed more cell death than VEH-MMSCs 9 h after UVB light exposure. Although at 24 h, VEH-MMSCs also presented death-related cell debris, the levels of cell debris were markedly enhanced in EDC-MMSCS. As expected, EDC-MMSCs presented a significantly decreased ability to repair the UVB-induced CPD compared with the control group at 6 h (VEH-MMSCs: 23.7% ± 2.5 vs. EDC-MMSCs: 8.3% ± 4.7, p < 0.05) and 12 h after the UVB exposure (VEH-MMSCs: 76.3% ± 4.1 vs. EDC-MMSCs: 7.6% ± 15, p < 0.05) (Fig. 3B).

### The transforming growth factor-β1 (TGFβ1) pathway regulates NER in rat MMSCs

TGFβ1 signaling has been implicated in regulating NER in human immortalized keratinocytes (HaCaT) cells (Qiang et al., 2016), but the link between these two pathways has not been evaluated in MMSC. We hypothesized that TGFβ1 pathway activation will compromise NER-dependent DNA repair in the healthy MMSC while TGFβ1 signaling inhibition will revert NER impairment in EDC-MMSC. To verify this hypothesis, we treated VEH-MMSCs with exogenous TGFβ1 and we observed that this activation suppressed CPD repair at 6 h (VEH-MMSCs: 70.6% ± 2.9 vs. VEH-MMSCs + TGFβ1: 33.3% ± 11.4, p < 0.05) and at 12 h (VEH-MMSCs: 71.3% ± 2.1 vs. VEH-MMSCs + TGFβ1: 63.1% ± 1.2, p < 0.05) after UVB light exposure (Fig. 3C). In this sense, when we inhibited TGFβ Receptor I on EDC-MMSCs we found that EDC-MMSCs recovered the capacity to repair CPD at 6 h (EDC-MMSCs: 25.9% ± 6.7 vs. EDC-MMSCs + TGFβ RI inhibitor: 53.4% ± 7, p < 0.05) (Fig. 3D). This data implies that TGFβ1 pathway is involved in regulating NER pathways in rat MMSCs.

### Uvrag gene expression is affected by TGFβ1 activation and inhibition on rat MMSCs

**Figure 4:**
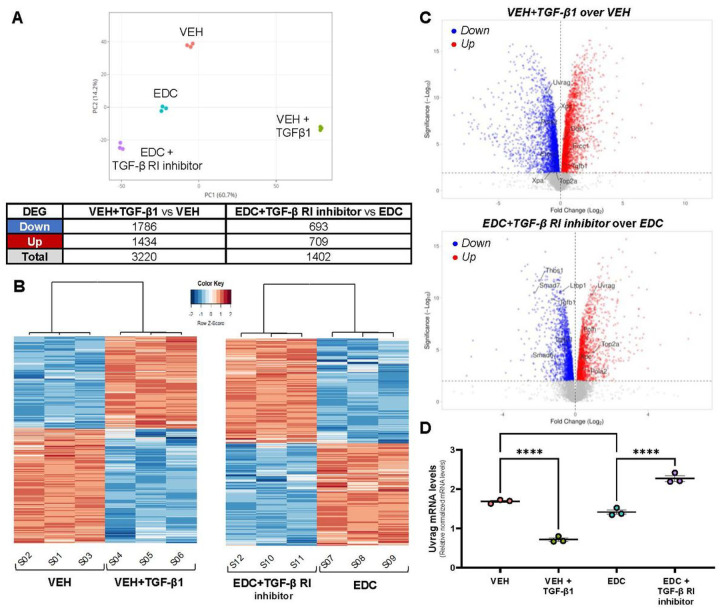
Effect of TGFβ1 pathway activation and inhibition on gene expression of rat VEH- and EDC- MMSCs. **A**) Principal component analysis plot showing samples clustering and DEGs table (Fold change ≥ 1.5, FDR of 0.05). **B**) Heatmaps representing the DEGs clustered using Pearson correlation in VEH-MMSCs treated with vehicle or TGFβ1 (10 ng/ml) for 48 h (left), and EDC-MMSCs treated with vehicle or TGFβ Receptor I inhibitor (2μM) for 24 h (right). Data is scaled by Z-score for each row. **C**) Volcano plots showing genes downregulated (blue dots) or upregulated (red dots) statistically significant. **D**) mRNA levels of *Uvrag* in VEH MMSCs treated with vehicle or TGFβ1 (10 ng/ml) for 48 h, and EDC MMSCs treated with vehicle or TGFβ Receptor I inhibitor (2μM) for 24 h. *18s* was used to normalize the expression data. Data are shown as mean ± S.E.M. *= p< 0.05, ****= p< 0.0001

**Figure 5:**
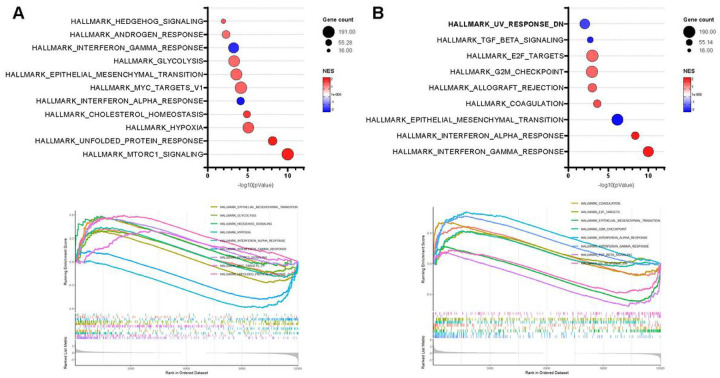
Effect of TGFβ1 pathway activation and inhibition on pathway enrichment of rat VEH- and EDC- MMSCs. A bubble chart of the Gene Set Enrichment Analysis (GSEA) (top) and enrichment plot (bottom) using the Hallmark MSigDB collection for **A**) VEH-MMSCs treated with vehicle or TGFβ1, and **B**)EDC-MMSCs treated with vehicle or TGFβ Receptor I inhibitor comparisons. Normalized enrichment score (NES) is a metric whose sign corresponds to which end of the dataset is enriched in the tested gene set

We performed RNA-seq analysis to further investigate the effect of TGFβ1 activation and inhibition in VEH and EDC-MMCs, respectively. The principal component analysis indicated that samples clustered by group (Fig. 4A). A total of 3220 DEG were found in the comparison of VEH-MMSCs treated with vehicle or TGFβ1. On the other hand, we observed 1402 DEG in EDC-MMSCs treated with vehicle or TGFβ Receptor I inhibitor (Fig. 4A). The heatmaps confirmed that samples were separated by treatment (Fig. 4B). Volcano plots in Fig. 4C illustrate the distribution of DEG in VEH-MMSCs treated with vehicle or TGFβ1 (top) and in EDC-MMSCs treated with vehicle or TGFβ Receptor I inhibitor (bottom). Genes of interest involved in NER and TGFβ1 pathways are indicated in the volcano plots. Interestingly, we observed that *Xpa* and *Xpc*, two NER members that were downregulated in EDC- compared to VEH-MMSCs (Fig. 2), were also downregulated on VEH-MMSC after the treatment with exogenous TGFβ1 (Fig. 4C, top). In addition, the *Ddb1* gene, which showed increased mRNA and protein levels in EDC-compared to VEH-MMSCs (Fig. 2), presented the same outcome in TGFβ1-treated VEH-MMSCs compared to the control (Fig. 4C, top). However, *Xpf* (also known as *Ercc1*) showed the opposite result, since its levels were decreased in EDC- compared to VEH-MMSCs (Fig. 2) but increased in VEH-MMSC after the TGFβ1 treatment (Fig. 4C, top). Regarding the effect of TGFβ Receptor I inhibitor on EDC-MMSCs transcriptome, we observed that the treatment increased the levels of *Xpc* gene (Fig. 4C, bottom), which, as we mentioned above, were downregulated in EDC-compared to VEH-MMSCs (Fig. 2). It is important to highlight that, as expected, the treatment of EDC-MMSCs with TGFβ Receptor I inhibitor downregulated several genes that belong to TGFβ1 signaling such us *Thbs1, Ltbp1, Tgfb1, Tgfbr1, Smad6 and Smad7* (Fig. 4C, bottom). Furthermore, UV-radiation Resistance Associated Gene (*Uvrag*) expression was downregulated in VEH-MMSCs after the treatment with exogenous TGFβ1 and upregulated after TGFβ Receptor I inhibition in EDC-MMSCs. *Uvrag* specifically interacts with DDB1, which together with DDB2, checks the whole genome for damage independently of transcriptional status [44]. We confirmed the results by qPCR (Fig. 4D), noting that *Uvrag* mRNA levels were decreased in EDC compared to VEH-MMSCS. Next, we analyzed the top enriched Hallmark gene sets in VEH-MMSCs treated with TGFβ1versus vehicle (Fig. 5A) and EDC-MMSCs treated with TGFβ Receptor I inhibitor or vehicle (Fig. 5B) by gene set enrichment analysis (GSEA) using Hallmark biological processes. The most significant enriched pathway in VEH-MMSCs treated with TGFβ1 in comparison with vehicle was HALLMARK_MTORC1_SIGNALINGIG (Fig. 5A, top) while in EDC-MMSCs treated with TGFβ Receptor I inhibitor versus vehicle was HALLMARK_INTERFERON_GAMMA_RESPONSE (Fig. 5B, top). Interestingly, GSEA identified that HALLMARK_UV_RESPONSE_DN gene set were significantly altered in EDC-MMSCs after TGFβ Receptor I inhibitor (Fig. 5B). Altogether, these results confirm the link that exists between TGFβ1 and NER pathways.

## Discussion

Transforming growth factor beta (TGFβ) is a multipotent cytokine that is involved in several pathological processes in many cell types. Misregulation of TGFβ activity has been related to tumorigenesis [45], including the development of UFs [46]. Recent studies have identified a connection between EDCs and TGFβ. Song et al. [47] have shown that bisphenol S (BPS), an industrial EDC, increases the mRNA and protein levels of TGFβ in non-small cell lung cancer (NSCLC) cells, and that their upregulation mediates BPS-induced NSCLC cell migration. Interestingly, in 3D human uterine leiomyoma (ht-UtLM) spheroids, the treatment with tetrabromobisphenol A (TBBPA), a derivative of bisphenol A (BPA), were found to induce an upregulated expression of profibrotic genes and corresponding proteins associated with the TGFβ pathway [32]. The previously mentioned studies focused on the direct effects of EDC exposure on TGFβ signaling, but it is likely that both direct and developmental EDC exposure may be mediated through similar pathways. In this sense, BPA exposure in pregnant rats delayed bone development and reduced bone mass in female offspring, and these results were accompanied by downregulated TGFβ signaling pathway in the bone tissue [48]. In contrast, maternal exposure to di-n-butyl phthalate (DBP), which induces renal fibrosis in adult rat offspring, is related to increased TGFβ mRNA and protein levels in the kidneys of DBP-exposed compared to unexposed 18-months old offspring [49]. In this current work, we have observed that EDC-MMSCs presented increased mRNA and protein levels of LTBP1, THBS1, and TGFβ1, and higher secreted levels of TGFβ1 compared to VEH-MMSCs, concluding that developmental EDC exposure overactivated TGFβ1 pathway. In the study by Liu et al. [32], TBBPA treatment activated TGFβ signaling through phosphorylation of TGFβR1 and downstream effectors SMAD2 and SMAD3 in a 3D ht-UtLM spheroid model. Similarly in our study, we have detected increased levels of SMAD2 phosphorylation confirming the downstream activation of TGFβ pathway, which after oligomerization with SMAD4, binds the DNA to mediate transcriptional activation or repression of target genes [50].

Besides the capacity of the EDCs to mimic the action of endogenous hormones, they have also been reported to exert genotoxic and mutagenic effects [51, 52]. The exposure to EDCs *in uterus* or during early life is associated with the progression of diseases later in life [53, 54]. Developmental periods present increased susceptibility to environmental stressors and these components become important risk factors for adverse health outcomes. In this sense, epidemiological studies have suggested associations between several EDCs and increased UF prevalence and severity [55–58]. In addition, experimental animal studies have shown evidence that early-life exposure to EDCs, such as DES or genistein, induces anatomic abnormalities in the reproductive tract, including uterine tumors [59, 60]. It is important to highlight that environmental exposure to EDCs also can reprogram the cell epigenome, resulting in gene expression changes [6].

Several studies have demonstrated the presence of MMSCs [61, 62], which are able to self-renew while producing daughter cells that differentiate, and are susceptible to reprogramming by EDC exposure. Evidence points out that EDC or their reactive intermediates can interact with DNA altering DNA bases and leading to DNA damage [51, 63]. Among the most common DNA lesions, single- and double-strand breaks [64], oxidative damage [65] and DNA adducts formation [66, 67] have been reported. Moreover, EDC can act through epigenetic mechanisms by which DNA damage repair is altered. The incapacity to correctly repair the DNA damage provoked by these compounds can lead to mutations and consequently, cells undergo modifications resulting in tumorigenesis. Moreover, the accepted model for the development of UFs establishes that they originate from an abnormal MMSC that acquire a driver mutation in pivotal genes such as *TSC-2* in the Eker rat or *MED12* in women. In particular, the Eker rat (*TSC-2*^Ek/+^), inactivation of the wild-type *TSC-2* allele commonly occurs by loss of heterozygosity (LOH, caused by direct deletion, deletion due to unbalanced rearrangements, etc.). However, other mechanisms such as point mutation have also been reported [68]. Here we showed that MMSCs isolated from Eker rats that were exposed in early life to DES, a potent EDC, presented lower nucleotide excision repair (NER) capacity compared to VEH-MMSCs. Importantly, defective NER causes the accumulation of point mutations and genomic instability [69]. Therefore, this impaired capacity to repair the DNA could lead to the loss of the wild-type *TSC-2* allele in EDC-MMSCs, which would trigger the development of uterine tumors at a higher frequency in EDC-exposed Eker rats than unexposed. We found that the observed decreased NER capacity could be related to lower mRNA and protein levels of several members of this pathway, such as XPC, an indispensable factor for the initial recognition of bulky DNA damage [70]. There is evidence that demonstrates that the availability and the activity of NER factors can be regulated by environmental factors. In this sense, Notch et al. [71] described a marked reduction in the expression of genes involved in the NER pathway, including XPC, in the livers of zebrafish exposed to the xenoestrogen 17-α-ethinylestradiol (EE2). This study expanded our previous findings showing that developmental EDC exposure decreased DNA end-joining ability [22], and impaired ability to repair DNA double-strand breaks (DSBs) by homologous recombination pathway [21] in rat MMSCs.

Furthermore, we showed that increased levels of TGFβ1 affected the repair of bulky DNA damage, through modulation of nucleotide excision repair (NER). Although contradictory, several works showed a connection between TGFβ1 and DNA damage. While some authors showed that the inhibition of TGFβ pathway tends to mitigate DNA damage responses and increase genomic instability [72–74], others demonstrated a possible role of activated TGFβ signaling in reducing the expression and/or activity of some genes involved in DNA repair [29, 75, 76]. Regarding TGFβ1 and NER pathway, Qiang et al. [29] have shown that the activation of the TGFβ pathway impairs UV-induced DNA repair by suppressing the transcription of XPC and DDB1. Additionally, we demonstrated that the treatment of VEH-MMSCs with exogenous TGFβ1 led to a decreased repair of DNA damage formed by ultraviolet-B radiation; a comparable response to that observed in EDC-MMSCs, which constitutively present activated endogenous TGFβ1 signaling. In accordance with this, we have shown that EDC-MMSCs treated with TGFβ RI inhibitor recover the capacity to repair the DNA damage after 6h of UVB light exposure. Furthermore, we use RNA-seq data to identify NER-related genes that are affected by activation or inhibition of TGFβ pathway in MMSCs. In particular, we discovered that TGFβ signaling regulates the expression of genes such as *UVRAG*, which is essential in the NER pathway.

EDC exposure may be one factor driving the observed disparities in UF burden between Black and White women [77], as studies suggest that exposure to certain environmental EDC is higher in non-whites populations [78]. In this sense, a systematic review published by Ruiz et al. affirms that Black, Latinos, and low-income individuals have greater exposure rates to EDCs, such as certain phthalates, BPA, polychlorinated biphenyls, and organochlorine pesticides, than any other ethnic and sociodemographic population groups [79]. Importantly, it is essential to recognize modifiable sources of EDC exposure that bring opportunities for reduction in racial/ethnic health disparities.

**Figure 6:**
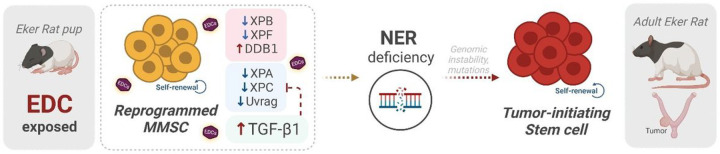
Early life exposure to endocrine-disrupting chemicals reprograms rat MMSC and impaired their capacity to repair the DNA. Myometrial stem cells (MMSC) from Eker rats exposed to endocrine disrupting chemicals (EDCs) in early life present lower levels of several members of nucleotide excision repair (NER) pathway, and overactivation of the TGFβ pathway which is also linked to changes in this DNA damage repair signaling. The reprogrammed MMSCs present impaired NER capacity, leading to increased genetic instability, arise of mutations, and their transformation into tumor-initiating stem cells, which would result in uterine tumorigenesis later in life. Created with BioRender.com

In summary, our findings pinpoint a link between the overactivation of TGFβ and the impaired ability of rat MMSCs to repair DNA damage through the NER pathway, and both provoked by the developmental exposition to EDC, which have implications for uterine tumor development (Fig. 6).

## Figures and Tables

**Table 1:** Rat primer sequences for RT-qPCR.

Symbol/Alias	Gene	Forward primer sequence (5′−3′)	Reverse primer sequence (5′−3′)
*Thbs-1*	Thrombospondin 1	TCGGGGCAGGAAGACTATGA	ACTGGGCAGGGTTGTAATGG
*Smad2*	Mothers against decapentaplegic homolog 2	GGGAAGTGTTTGCCGAGTG	AGCCTGGTGGGATTTTGC
*Xpa*	DNA damage recognition and repair factor	CAGACACCAGAGCCACTTTAC	GCAGACACCCATACACAATGA
*Xpb, Ercc3*	Xeroderma pigmentosum complementation group B, ERCC excision repair 3, TFIIH Core Complex Helicase Subunit	GGGTACTCAGAGCCAAGAAAG	GAATCTCTGTCGCTTGGTAGAA
*Xpc*	Xeroderma pigmentosum complementation group C	CACCTCCATCAGCACATACAA	ACAGCTTCTCCACGACAATAC
*Xpf, Ercc4*	Xeroderma pigmentosum complementation group F, ERCC Excision Repair 4, Endonuclease Catalytic Subunit	TAAGCTCACACTCCTCACCT	CCAGGGTTATACCTGTCTGA
*Xpd, Ercc2*	Xeroderma pigmentosum complementation group D, ERCC excision repair 2, TFIIH core complex helicase	TTACTACAGCGCAGAGCCAG	ACCCCAAACATTTCACCCACT
*Ddb1*	DNA damage-binding protein 1	CACGGTTCCTCTCTATGAATCTC	TAGTGCCTCCACTGGTATCT
*Ddb2*	DNA damage-binding protein 2	TGGTGGTTACAGGAGACAATATG	GCCACATGGGCTACTTTCT
*18S*	18S ribosomal RNA	CACGGACAGGATTGACAGATT	GAGTCTCGTTCGTTATCGGAATTA

## Data Availability

The datasets (Raw FASTQ files) generated during and/or analyzed during the current study are available in the NCBI Gene Expression Omnibus database with accession number GSE157503 (Supplementary Figure 1 - VEH-MMSC and EDC-MMSC) and GSE225636 (Figures 4 and 5- VEH-MMSC, VEH-MMSC +TGFβ1, EDC-MMSC, and EDC-MMSCs + TGFβ RI inhibitor).
